# Exposure of patients with chronic kidney disease on dialysis to pesticides

**DOI:** 10.1590/2175-8239-JBN-2022-0030en

**Published:** 2022-09-05

**Authors:** Greice Kelli de Medeiros Martins, Nathália Cervo Pereira, Natália Veronez da Cunha, Lenita Agostinetto

**Affiliations:** 1 Centro de Terapia Renal, Clínica de Hemodiálise, Lages, SC, Brasil.; 2 Universidade do Planalto Catarinense, Lages, SC, Brasil.; 3 Universidade do Planalto Catarinense, Programa de Pós-Graduação em Ambiente e Saúde, Lages, SC, Brasil.

**Keywords:** Pesticides, Kidney Diseases, Life Style, Praguicidas, Nefropatias, Estilo de Vida

## Abstract

**Introduction:**

Pesticides can trigger kidney disease.

**Objective:**

To describe the exposure to pesticides of patients with chronic kidney disease on dialysis.

**Methods:**

Quantitative and descriptive field research, with 90 patients with chronic kidney disease on dialysis in two hemodialysis units in the state of Santa Catarina, through the application of a structured questionnaire. Participants were divided into two groups: with and without exposure to pesticides. The questionnaire was applied in hemodialysis clinics during treatment. Laboratory test values were collected from clinical records. Data were analyzed using descriptive statistics and association using the chi-square test. For laboratory test data, a comparison of means was performed using the unpaired Student’s t-test between the groups.

**Results:**

The mean age of exposed participants was 58 years (±13.7; minimum = 23; maximum = 75) and that of non-exposed participants was 64 years old (±13.9; minimum = 35; maximum = 96). Of the 90 patients, 30% were exposed to pesticides. The mean exposure time was 6.7 ± 3.8 hours/day. There was a statistically significant association between the preparation of the mixture with pesticides and diabetes (p ≤ 0.048). There was no statistically significant difference between the results of laboratory tests in the exposed and non-exposed groups.

**Conclusion:**

This study shows that pesticides can be triggering factors for chronic kidney disease (CKD); however, we must expand research in this field to prove the relationship between exposure to pesticides and CKD.

## Resumo

**Introdução:**

O uso de agrotóxicos pode desencadear doença renal.

**Objetivo:**

Descrever a exposição a agrotóxicos de pacientes com doença renal crônica em tratamento dialítico.

**Métodos:**

Pesquisa de campo, quantitativa e descritiva, com 90 portadores de doença renal crônica em tratamento dialítico em duas unidades de hemodiálise no estado de Santa Catarina, por meio da aplicação de um questionário estruturado. Os participantes foram divididos em dois grupos: sem e com exposição a agrotóxicos. O questionário foi aplicado nas clínicas de hemodiálise durante o tratamento. Foram coletados valores de exames laboratoriais dos prontuários clínicos. Os dados foram analisados pela estatística descritiva e associação pelo teste qui-quadrado. Para os dados dos exames laboratoriais, foi realizada comparação de médias pelo teste t de Student não pareado entre os grupos.

**Resultados:**

A idade média dos participantes expostos foi de 58 anos (±13,7; mínimo = 23; máximo = 75) e a dos não expostos, de 64 anos (±13,9; mínimo = 35; máximo = 96). Dos 90 pacientes, 30% foram expostos a agrotóxicos. O tempo médio de exposição foi de 6,7 ± 3,8 horas/dia. Houve associação estatística significativa entre o preparo da calda com agrotóxicos e a presença de diabetes (p ≤ 0,048). Não houve diferença estatística significativa entre os resultados dos exames laboratoriais do grupo exposto e do não exposto.

**Conclusão:**

Esta pesquisa evidencia que os agrotóxicos podem ser fatores desencadeadores da doença renal crônica (DRC), entretanto sugere-se ampliar pesquisas na área que possam comprovar a relação entre exposição a agrotóxicos e DRC.

## Introduction

Chronic kidney disease (CKD) consists of kidney damage with progressive and irreversible loss of kidney function^1^. The main factors causing a drop in glomerular filtration rate are chronic diseases such as diabetes and hypertension; in addition, several chemicals also called xenobiotics can lead to kidney dysfunction^2^.

CKD is an important medical and public health problem in both developed and developing countries^3^. It is estimated that CKD affects more than 10% of the world’s adult population^4^. In Brazil, the prevalence of patients on a chronic dialysis program has more than doubled in the last eight years^5^. In the state of Santa Catarina, 2,541 cases of chronic kidney disease were recorded in 2016, according to data from the State Department of Health (2018)^6^.

Brazil is a leading country in agricultural production, and also the world’s largest consumer of pesticides. There are regions in the country where the use of pesticides is more intense due to the types of crops and size of the cultivated area; therefore, the exposure and the risk of intoxication are higher. For example, in Mato Grosso, Goiás and Mato Grosso do Sul there is extensive production of soy, which is currently the crop that most consumes pesticides in Brazil^7^. In Santa Catarina, there is also intense use of pesticides, but the use is directed to apple cultivation mainly, in which an average of 35 applications per crop can occur^8^.

Santa Catarina’s agriculture is developed with high technological levels, applied in intensive production systems and with high added value^9^; despite this, agricultural production in the region is based on conventional cultivation with the use of pesticides for the management of agricultural crops. The expressive and frequent use of pesticides causes damages with environmental and human contamination^10,11^. In relation to human health, the harmful effects of the use of pesticides are diverse and can cause acute or chronic poisoning^12^. In Santa Catarina, the 2018 National Health Surveillance Report for Populations Exposed to Pesticides showed that, in 2015, there were 695 notified cases^6^. Studies also point to cases of acute poisoning in the Serra region of Santa Catarina^8,13^. Research has shown that several chemical groups of pesticides can develop the main risk factors that cause chronic kidney disease or directly affect the kidneys^2,13–16^. In general, pesticides can trigger CKD directly and indirectly, or even through the effect of heat stress on farmers due to the ergonomics of Personal Protective Equipment (PPE) combined with insufficient fluid intake, which leads to body water depletion and consequent harm to kidney health^14^.

Considering that the use of pesticides in Brazil has been growing at an alarming rate in the last decade, and the number of chronic kidney patients is growing at the same time, the objective of this study was to describe the exposure to pesticides of patients with chronic kidney disease on dialysis.

## Methods

This was a quantitative and descriptive study, that took place in two kidney therapy clinics in the state of Santa Catarina: hemodialysis clinic Centro de Terapia Renal de Lages, SC, and Clínica de Hemodiálise de Videira, SC.

All the participants in the study were patients with stage-5 CKD undergoing dialysis during the study period (July 2020 to March 2021), who met the inclusion criteria: undergoing dialysis treatment (stage 5) in the clinics in the cities of Lages and Videira, they were 18 years of age or older and agreed to participate in the study of their own free will, signing the Free and Informed Consent Form (FICT). Patients who did not accept to participate in the study and those who did not have the physical and/or psychological conditions to be part of the study – evaluated by the nephrologist – were taken off.

Sixty-three patients were investigated at the clinic in Lages and 27 patients at the clinic in Videira, totaling 90 patients, and the technique used to choose the participants was intentional.

Data collection was performed through the application of a questionnaire, analysis of medical records and collection of blood-test results from patients.

The questionnaire, prepared by the team of researchers and divided into two stages, consisted of closed and some open questions. The first step was answered by all the study participants. The first part consisted of: identification of some sociodemographic data; investigation of the participants’ lifestyle, such as diet, physical activity, dependence on alcoholism and smoking, water consumption and relationships; and gathering information on the etiological factors that can lead to the development of CKD, with questions about possible exposure to pesticides, other xenobiotics that are not pesticides and the presence of comorbidities. Such categorization made it possible to divide the study participants into two groups: without exposure to pesticides (control group) and with exposure to pesticides. For the exposed group, only exposure to pesticides was considered and not to other xenobiotics.

The second stage of the questionnaire was answered only by participants who claimed to have been exposed to pesticides at some point in their lives. The research participant’s association with agriculture and possible lifetime exposure to pesticides was explored. For this, questions were elaborated that addressed: time and place of work with agriculture, use of pesticides, types of pesticides used, way of handling pesticides, use of personal protective equipment (PPE), exposure to pesticides, among other information.

The questionnaire was administered to the patients by the researchers at the dialysis care centers during the hemodialysis session. The questionnaire was read by the researcher and answered by the interviewee, and the researchers recorded the answers given by the interviewees. The application of the questionnaire lasted an average of 30 minutes.

The clinical records of the CKD patients were also analyzed, in which the results of tests that are already part of the clinical evaluation were collected, such as levels of urea, creatinine, transaminase, potassium, calcium and phosphorus.

The study started after approval by the Research Ethics Committee, approved according to protocol number 4,073,680. Before starting the study with the participants, the informed consent was read to those in the study and only continued with those participants who agreed with the study and signed the document.

Data were analyzed using descriptive statistics with frequency, mean and standard deviation of the mean. For categorical data, a variable association test was performed using the chi-square test. For numerical data, the Shapiro-Wilk normality test was initially performed; with confirmed normality, the means of the control group (without exposure to pesticides) and those of the group with exposure to pesticides were compared using the unpaired Student’s t-test. The significance level adopted was p < 0.05. Data were processed and analyzed using the SPSS 2.0 statistical program.

## Results

The mean age of the exposed participants in this study is 58 years (±13.7; minimum = 23; maximum = 75) and that of the unexposed, 64 years (±13.9; minimum = 35; maximum = 96). Most participants were male, both exposed and not exposed to pesticides (66.7%), married (29.6% of the exposed group and 61.9% of the non-exposed group) and have completed elementary school (48.1% of those exposed and 53.9% of those not exposed).

Regarding the lifestyle of the patients, most of them did not perform physical exercises, both the participants in the group exposed to pesticides (77.8%) and the participants in the group of those not exposed (85.7%). Most did not have any leisure activity (55.6% and 57.1% of those exposed and not exposed, respectively) (Supplementary material). Of those who reported having leisure activities, the most mentioned were fishing, walking, traveling, watching television, reading, making handicrafts, among others.

Most of the participants in both groups reported taking continuous use medication (85.2% and 93.7% of exposed and unexposed individuals, respectively); and in both groups the majority did not smoke (63.0% and 49.2% of exposed and non-exposed individuals, respectively) (Supplementary material).

As for eating habits, the majority reported low salt intake in both groups (77.8% and 84.1% of those exposed and unexposed, respectively), consumption of fatty foods (92.6% and 92.1% exposed and unexposed, respectively) and sweets (88.9% and 92.1% of exposed and unexposed, respectively) up to three times a week and consumption of alcoholic beverages only once a week (92.6% and 92.1% of exposed and unexposed, respectively) (Supplementary material). In addition, on average, patients exposed to pesticides consume 1 liter of water/day (±0.7; minimum = 0.2 L; maximum = 3 L) and non-exposed patients consume an average of 0.5 liter of water/day (±0.7; minimum = 0.2 L; maximum = 3 L).

As for relationships, the majority of both groups reported good family life (92.6% and 98.4% of those exposed and not exposed, respectively), with neighbors (85.2% and 95.2% of the exposed and unexposed, respectively), with the doctor (92.6% and 100.0% of exposed and unexposed, respectively) and with society in general (85.2% and 96.8% of exposed and non-exposed patients, respectively). (Supplementary material).

Regarding comorbidities in the exposed group, most patients did not have diabetes (81.5%), and all of them underwent some type of treatment; in the non-exposed group, most had diabetes (60.3%), and most of those who had the disease were under some treatment (50.8%) (Supplementary material). The most cited treatments were: NPH insulin, glibenclamide, metformin, empaglifozin, linagliptin and glyciphage. Most patients were hypertensive, both those in the group exposed to pesticides (70.4%) and those not exposed (84.1%); and the majority for both cases were on medication for hypertension (Supplementary material), and some of the drugs used were: amlodipine, clonidine, enalapril, losartan, hydrochlorothiazide, nifedipine, atenolol, olmesartan. As for the presence of hypertriglyceridemia, most patients in both groups did not have it (81.5% and 52.4% of exposed and non-exposed patients, respectively). Of those exposed, who had it, 7.4% were on treatment, and 33.3% of those not exposed were on treatment (Supplementary material), with simvastatin being the most used drug. Regarding lithiasis, the majority in both groups (92.6% and 95.2% of exposed and non-exposed individuals, respectively) did not have it, and among those who did, most did not undergo treatment (Supplementary material). The majority also did not have a urinary tract infection (55.6% and 65.1% of exposed and unexposed individuals, respectively); however, the minority of those who did, in both groups, underwent some treatment (Supplementary material), such as the use of antibiotics or surgery.

As for the risk factors associated with chronic kidney disease, of the 90 patients, 27 (30%) had been exposed to pesticides, and 23 (25.6%) had been exposed to some type of xenobiotic (Table 1), such as sulfuric acid, ammonia, heavy metals, paint thinner, petroleum products and liquid LPG. None of the participants reported using pesticides for household use or pesticides for veterinary use. The mean exposure duration to xenobiotics was 28 years (±17.3; minimum = 4 years; maximum = 50 years). In addition, 84 (93.3%) used continuous medication and 81 (90.0%) had some type of comorbidity (hypertension, diabetes, etc.).

**Table 1.** Risk factors associated with dialysis patients in the study from the cities of Lages and videira, SC, 2020

Variables	n	%
**Exposure to pesticides**		
Yes	27	30.0
No	63	70.0
**TOTAL**	**90**	**100**
**Exposure to xenobiotic agents**		
Yes	23	25.6
No	67	74.4
**TOTAL**	**90**	**100**
**Continuous use of medication**		
Yes	84	93.3
No	6	6.7
**TOTAL**	**90**	**100**
**Comorbidities**		
Yes	81	90.0
No	9	10.0
**TOTAL**	**90**	**100**

Source: the authors.

**Chart 1.**
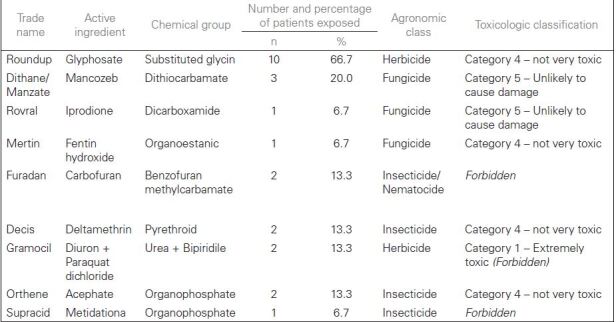
Main types of pesticides to which the study participants reported exposure to

Source: the authors. *The total n considered was 15 patients among the 27 exposed, because the others did not remember the name of the pesticide used.

Of the 27 patients who had already been exposed to pesticides, three of them (11.1%) were still working in agriculture, and those who stopped working did so for an average of 11.4 years (±11.9; minimum = 0.5 years ; maximum = 45 years). When the exposed were asked about the categories of agricultural crops they worked or had worked with, the responses were grains (19.2%), fruits (38.5%), vegetables, tobacco (3.8% for each category, respectively) and more than one category (34.6%). The main types of pesticides to which the participants were exposed to are shown in Chart 1.

Table 2 describes the main forms of exposure to pesticides.

**Table 2.** Exposure to pesticide by dialysis patients participating in the study from the cities of lages and videira, SC, 2020. N = 27

Variables	n	%
**Currently uses pesticides?**		
Yes	5	18.5
No	22	81.5
**Currently prepares the syrup?**		
Yes	2	7.4
No	25	92.6
**Ever prepared the syrup anytime in life?**		
Yes	9	33.3
No	16	59.3
Does not apply	2	7.4
**Currently applies it?**		
Yes	3	11.1
No	24	88.9
**Ever Applied during your life?**		
Yes	12	44.4
No	12	44.4
Does not apply	3	11.1
**Place where the pesticide was prepared?**		
Garden/plantation	8	29.6
Dedicated shed	15	55.6
No defined place	1	3.7
Both	1	3.7
Other	2	7.4
**Type of spraying?**		
Tractor	14	51.9
Backpack pump	8	29.6
Both	5	18.5
**Application tractor has protective booth?**		
Yes	3	11.1
No	16	59.2
Does not apply	8	29.6
**Owns PPE?**		
Yes	11	40.7
No	16	59.3
**Wears PPE during syrup preparation?**		
Yes	10	37.0
No	17	63.0
**Wears PPE during the application?**		
Yes	10	37.0
No	17	63.0
Variables	n	%
**Follows order to wear PPE?**		
Yes	3	11.1
No	8	29.6
Has no PPE	16	59.2
**Follows order to remove PPE?**		
Yes	3	11.1
No	8	29.6
Has no PPE	16	59.2
**Underwear underneath the PPE has been wet by pesticides?**		
Yes	11	40.7
No	0	0.0
Has no PPE	16	59.3
**When the underwear got wet, what did you do?**		
Continued the work, then changed it	8	29.6
Changed immediately	3	11.1
Has no PPE	16	59.3
**Washes the PPE?**		
Yes	9	33.3
No	18	7.4
Has no PPE	16	59.3
**Destination of the PPE underwear?**		
Changes and places together with the remaining dirt clothes	3	11.1
Washes separately	8	29.6
Does not have a PPE	16	59.3
**Has meals near the place Where you handle the pesticide?**		
Yes	4	14.8
No	23	85.2
**Smokes near the area of pesticide handling?**		
Yes	1	3.7
No	18	66.7
Does not smoke	8	29.6

PPE = Personal Protection Equipment. Source: the authors.

It is noteworthy that, currently, the majority no longer use pesticides (81.5%) (Table 2), and on average stopped using pesticides 10.4 years ago (±9.8; minimum = 1 year; maximum = 45 years). In addition, these participants reported that they had used pesticides for an average of 14.4 years (±12.9; minimum = 1.5 years; maximum = 50 years). However, the other 18.5% continued to use it (Table 2). It is noteworthy that the exposed patients (n = 27) were in stage 5 of CKD and on hemodialysis for an average of 3 years (±2; minimum = 0.5 years; maximum = 9 years), indicating that the exposure preceded the outcome.

The mean exposure time reported by exposed patients was 6.7 hours daily (±3.8; minimum = 1 hour; maximum = 16 hours). Few (7.4%) still prepared pesticide syrup and 33.3% had already prepared it at some point in their lives (Table 2). Among those who had already prepared, this occurred on average for 10.6 years (±8.8; minimum = 2 years; maximum = 30 years).

Few also currently used pesticides (11.1%). However, another 44.4% had already applied at some point in their lives, on average for 11.1 years (±9.3; minimum = 2 years; maximum = 30 years). Most patients (66.7%) reported that the distance between the pesticide storage place and their residence is/was greater than 30 meters; however, 33.3% reported that it is/was less than this distance. As for the preparation of pesticides, the majority (55.6%) reported that it is/was done in a shed exclusively for this purpose, although there are still irregularities (Table 2). For the application of pesticides, most participants reported that they used/used a tractor (51.9%) and generally did not have/had a protective booth (59.2%) (Table 2).

The highest percentage (59.3%) of exposed participants stated that they did not wear personal protective equipment (PPE) (Table 2). Of those who had PPE, 37% wore it both for the preparation of the syrup and for the application of pesticides. However, most participants did not use full PPE, that is, most did not use a filter mask (77.7%), an unfiltered mask (88.9%), gloves (63.0%), boots (66.7%), apron (88.9%), visor (88.9%), water-repellent overalls (74.1%) and Arab cap or water-repellent hood (88.9%). In addition, part of the participants (29.6%) did not follow/followed the order to wear and remove the PPE (Table 2). It is noteworthy that all those who reported using/using PPE also reported that the underwear of the PPE was wet with pesticides, with 29.6% continuing to work and only later changing the wet clothes. The washing of these clothes is usually (29.6%) done separately from other people’s clothes in the household (Table 2).

As for the washing of PPE, the highest percentage of those who use/used (33.3%) reported that the individual washes the PPE himself and generally washes it every time he uses/used it or weekly. Finally, most patients did not eat (85.2%) or smoke (66.7%) when handling pesticides (Table 2).

There was a statistically significant association between the preparation of the mixture with pesticides and the presence of diabetes; that is, patients who reported that they had already prepared the pesticide syrup at some point in their lives also reported having diabetes (p ≤ 0.048). There was also a statistically significant association between the use of a tractor with a protective cabin and medication for continuous use, and patients who reported that they used a tractor for spraying pesticides without a protection cabin also reported that they currently used medication continuously (p ≤ 0.009). There was a significant association between the treatment given to clothes worn under PPE and the presence of hypertension; that is, patients who reported that they separated clothes contaminated with pesticides from other clothes when washing at home also reported that they did not have hypertension (p ≥ 0.046).

The results of laboratory tests (urea, creatine, transaminase, potassium, calcium and phosphorus) did not indicate changes, according to reference values for these patients. There was no statistical difference when comparing the results of laboratory tests of dialysis patients not exposed and those exposed to pesticides, as shown in Table 3.

**Table 3.** Comparing the laboratory tests of dialysis patients (mean and standard deviation) exposed and not exposed to pesticides from the cities of lages and videira, SC, 2020

Lab tests	Reference values^1^	Exposed	Not exposed	p
Urea (mg/dL)	10 a 50	127.9 ± 6.02 (n = 24)	129.4 ± 4.82 (n = 39)	0.85
Creatinine (mg/dL)	0.6 a 1.10	7.9 ± 0.63 (n = 24)	6.6 ± 0.43 (n = 39)	0.07
Transaminase (U/L)	TGO up to 31 TGP up to 32	19.0 ± 2.75 (n = 22)	21.8 ± 3.50 (n = 39)	0.52
Potassium (mEq/L)	3.5 a 5.5	5.2 ± 0.15 (n = 23)	5.3 ± 0.13 (n = 39)	0.59
Calcium (mg/dL)	8.6 a 10.3	8.9 ± 0.28 (n = 24)	9.0 ± 0.11 (n = 38)	0.51
Phosphorus (mg/dL)	2.5 a 4.5	6.1 ± 0.46 (n = 24)	5.4 ± 0.23 (n = 39)	0.16

Source: the authors. *The n from the lab tests of individuals exposed and those not exposed to pesticides correspond Only to that from those individuals who had the test available. ^1^ The reference values of the tests follow the standards from the Labhos lab, Lages, SC.

## Discussion

In general, it was evidenced in this research that 30% of the studied sample is exposed or was exposed to pesticides throughout their lives, as well as some statistical associations were found between exposure to pesticides with the presence of diabetes, hypertension and continuous use of medication. Research around the world indicates that pesticides can be considered precursor agents of chronic kidney disease^2,13–16^.

Regarding the characterization of the studied sample, there is a predominance of chronic kidney disease (CKD) in males, in the approximate age group of 60 years. Increasing age can cause renal atrophy and reduction of the renal cortex from the age of 30 onwards. Thus, aging can cause several changes in the renal system, such as atrophy, fibrosis, glomerular sclerosis, among others^17^.

Most research participants, from both groups, had only elementary education, which corroborates another study on the profile of dialysis patients^18^. Having completed higher education is a protective factor for CKD, as individuals with more favorable socioeconomic conditions, such as higher education, are less exposed to risk factors for the disease. In addition, these individuals usually have health insurance, and therefore greater access to tests and earlier diagnoses^19^.

Regarding lifestyle, research has shown that smoking is an important risk factor for triggering CKD. Elihimas Jr. et al.^20^ demonstrated the correlation between smoking as a risk factor for CKD progression. Inhalation of tobacco smoke produces several gases, some with nephrotoxic potential, such as heavy metals, which cause tubular toxicity, such as cadmium and lead^21,22^.

The prevalence of sedentary habits and the intake of foods rich in fats and sugars up to three times a week stand out as unfavorable practices. The lack of a physical exercise routine, and other habits, is directly related as a risk factor to CKD-based comorbidities, such as arterial hypertension and type 2 diabetes mellitus^19^.

In the presented sample, the highest percentage of dialysis patients had arterial hypertension, being the most prevalent pathology, and type 2 diabetes mellitus. In Brazil, about 63% of CKD cases are from patients with both comorbidities^23^. According to the Brazilian Census of Nephrology (2020), arterial hypertension is the main etiology of CKD^24^. These comorbidities require periodic monitoring and appropriate treatment; otherwise, they will contribute to a deleterious prognosis for patients with chronic kidney disease^25^.

A large part of the sample used medication for continuous use, primarily to control arterial hypertension. The damage caused by hypertension in chronic renal patients can be, among others: renal vasoconstriction – mainly of the preglomerular vasculature, microvascular damage, loss of peritubular capillaries, local ischemia, inability to excrete salt and hypertensive renal disease^26^.

Despite the most eminent causes of CKD, especially in developed countries, the described pathology is also related to occupational and environmental causes, including exposure to pesticides and other xenobiotics among the hypotheses, since certain pesticides commonly used in many parts in the world are recognized by the human body as nephrotoxic substances^2,13–15^.

Research in Brazil has shown cases of farmer intoxication by pesticides related to the occurrence of chronic diseases^27,28,29^. As shown by some studies in the mountainous region of Santa Catarina, where agriculture is one of the main economic drivers, there are deficiencies in the use and handling of these chemicals, as well as cases of acute intoxication and even associations with diseases^10,11^. An international study indicated an association between CKD and chronic exposure to specific pesticides^13^.

Most kidney patients in our study had been exposed to pesticides. Pesticides can affect kidney tissue from tubular cell toxicity or cause changes in renal blood flow, which leads to secondary tubular damage at a molecular level. In addition, in patients exposed to pesticides, the presence of large dysmorphic lysosomes in the proximal tubular cells of the nephrons was detected – very similar to what occurs in patients treated with calcineurin inhibitors after kidney transplantation^16^. Usually, such repercussion is conditioned to factors related to improper handling of substances, the high toxicity of certain products and the non-use or incorrect use of PPE^11,15^.

In this study, many did not have PPE, in addition, part of the respondents of the exposed group remained with PPE underwear wet with pesticides throughout the work period. PPE, despite not being 100% efficient in protecting against the toxic effects of pesticides, are essential to minimize the risk of acute and/or chronic poisoning^30^. It is understood that PPE still do not have ergonomic conditions, as research has shown that farmers who do not wear PPE report that they did not use it precisely because of the discomfort, difficulty breathing and heat caused by such equipment, among other arguments^11,31^, since PPE are not equipment designed for use by farmers but adapted from PPE for industrial use.

Among the factors that make pesticides possible triggers of CKD is the effect of heat stress due to the ergonomics of the PPE, combined with insufficient fluid intake by farmers during work or throughout life, as it can lead to body water depletion and, consequently, harm to kidney health^15^. Dehydration, heat stress and heat overload may be factors associated with agricultural work that could be related to CKD^32^.

In the present study, most patients exposed to pesticides had already prepared the mixture and sprayed pesticides at some time in their lives and applied the products by means of tractors without a protective cabin, which can further increase the risk of exposure, since the worker is more exposed to pesticide droplets that dissipate into the atmosphere after spraying^33^.

Although some countries regulate the need to use tractor cabins, as well as the type and way of using these cabins to protect themselves from contact with pesticides, in Brazil most tractors used in pesticide applications still do not have protection cabins, leaving the operators only to use the PPE as a way to avoid possible contamination by the substances^34^.

Among the categories most susceptible to the toxic effects of pesticides, agricultural workers and pesticide applicators are the most susceptible. In the present study, the average time of exposure to pesticides reported by patients was 6.7 hours. Based on these determinants, mainly on the chronological and physical component, on body water depletion, it is stated that daily work for more than 6 hours in the field under the sun is also an important modulator of nephrotoxicity^35^.

In our study, of the six main active ingredients used by patients, two of them, glyphosate and mancozeb, are correlated with the most widespread toxic substances throughout the national territory, emphasizing the concern with the indiscriminate use of these substances and their consequences^35^. In the scientific community, there is convincing evidence that exposure to glyphosate is a significant factor in CKD associated with the use of pesticides^36^. Thus, there is a likelihood with the chemical products used by exposed patients, evidencing that this herbicide may be a risk factor associated with kidney patients in this study.

There was also statistical evidence of the association between the preparation of the mixture with pesticides and diabetes, since patients who have already prepared the mixture with pesticides at some time in their lives are also carriers of diabetes. In Brazil, some authorized pesticides are associated with endocrine disruption, including the mancozeb, which exposure was reported by the study-patients as one of the pesticides to which they were exposed. In Brazil, populations exposed to these agricultural products tend to be more vulnerable to the onset of diseases related to the immune and endocrine systems, including diabetes^37^.

It is also noteworthy that patients in this sample were exposed to some other type of xenobiotics, in addition to pesticides, which is also a factor that may have contributed to the onset of CKD. In the present study, many patients reported exposure to heavy metals. Research has shown that these xenobiotics have the potential to cause kidney damage^38,39^. Nephrotoxic substances, such as heavy metals, paints and others, can cause various types of kidney damage with serious consequences, since the kidneys are the main route of organic excretion of such substances^40^. In this study, the intention was not to deepen the investigation of other xenobiotics, because the study was focused on pesticides only.

The lack of laboratory alterations in the study patients and the absence of differences in these exams of patients exposed to and not exposed to pesticides were already expected, since all of them undergo hemodialysis. During the process of dialysis, blood filtration occurs, that is, hemodialysis promotes the elimination of waste harmful to health, such as excess salt and fluids, as well as helps the body maintain the balance of substances such as potassium, urea and creatinine, among others^41^. Thus, from dialysis, excess fluid and toxins are removed from the blood of chronic kidney patients with the subsequent return of clean blood to the patient^41^.

Thus, patients exposed to pesticides may, from the dialysis process, have eliminated possible residues and toxins that may have remained in the body during exposure. Research has shown that the procedures currently used in nephrology may soon also be used more widely for the detoxification process of patients poisoned by pesticides^42^. This fact constitutes a probable argument for the lack of statistical difference between the laboratory tests of exposed and non-exposed patients in the present study.

In general, this study contributes to the technical, scientific and social fields in order to expand the dissemination of the risk to human health associated with the use and handling of pesticides. We also suggest this study should be expanded with humans and/or animals, mainly of an experimental nature, which can prove the relationship between exposure to pesticides and CKD, as well as the importance of other risk factors in the development of the disease.

## Supplementary Material

The following online material is available for this paper:

Table S1 – Lifestyles and comorbidities of dialysis patients (group exposed and not exposed to pesticides) study participants in the municipalities of Lages and Videira SC, 2020.
