# Supplementary material for: Exposure of patients with chronic kidney disease on dialysis to pesticides
Source: J Bras Nefrol. 2022 Sep 5;45(2):169–79. doi: 10.1590/2175-8239-JBN-2022-0030en (PMC10627121; doi:10.1590/2175-8239-JBN-2022-0030en)
Supplement: Table S1 [file 2175-8239-JBN-e20220030-pt-s1.pdf]

Braz. J. Nephrol. (J. Bras. Nefrol.)

https://doi.org/10.1590/2175-8239-JBN-2022-0030en

Supplementary Material to "Exposure of patients with chronic kidney disease on dialysis to pesticides"

Table S1 - Lifestyles and comorbidities of dialysis patients (groups Exposed and Not Exposed to Pesticides) participants of the study from the cities of Lages and Videira SC, 2020.

**Variables**	**Exposed**		**Not Exposed**	
**Physical exercise**	**n**	**%**	**n**	**%**
Yes	6	22.2	9	14.3
No	21	77.8	54	85.7
**TOTAL**	**27**	**100**	**63**	**100**
**Has leisure activities**				
Yes	12	44.4	27	42.9
No	15	55.6	36	57.1
**TOTAL**	**27**	**100**	**63**	**100**
**Takes medications**				
No	0	0.0	1	1.6
Continuous use	23	85.2	59	93.7
Controlled prescription	3	11.1	1	1.6
Continuous and controlled	1	3.7	2	3.2
**TOTAL**	**27**	**100**	**63**	**100**
**Smoker**				
Yes	3	11.1	10	15.9
No	17	63.0	31	49.2
Stopped smoking	7	25.9	22	34.9
**TOTAL**	**27**	**100**	**63**	**100**
**Lives with smoker**				
Yes	10	37.0	14	22.2
No	17	63.0	49	77.8
**TOTAL**	**27**	**100**	**63**	**100**
**Salt consumption**				
Does not	4	14.8	5	7.9
Little	21	77.8	53	84.1
Moderate use	2	7.4	5	7.9
**TOTAL**	**27**	**100**	**63**	**100**
**Fatty food intake**				
Up to three times per week	25	92.6	58	92.1

Variables	Exposed		Not Exposed	
More than three times/week	2	7.4	5	7.9
**TOTAL**	**27**	**100**	**63**	**100**
**Sweet intake**				
Up to three times/week	24	88.9	58	92.1
More than three times/week	3	11.1	5	7.9
**TOTAL**	**27**	**100**	**63**	**100**
**Alcohol intake more than once-a-week**				
Yes	2	7.4	5	7.9
No	25	92.6	58	92.1
**TOTAL**	**27**	**100**	**63**	**100**
**Friendly relationship with one's family**				
Good	25	92.6	62	98.4
Reasonable	2	7.4	1	1.6
**TOTAL**	**27**	**100**	**63**	**100**
**Friendly relationship with one's neighbors**				
Good	23	85.2	60	95.2
Reasonable	4	14.8	3	4.8
**TOTAL**	**27**	**100**	**63**	**100**
**Friendly relationship with one's physician**				
Good	25	92.6	63	100.0
Reasonable	2	7.4	0	0.0
**TOTAL**	**27**	**100**	**63**	**100**
**Friendly relationship with one's society**				
Good	23	85.2	61	96.8
Reasonable	4	14.8	2	3.2
**TOTAL**	**27**	**100**	**63**	**100**
**Has diabetes**				
Yes	5	18.5	38	60.3
No	22	81.5	25	39.7
**Treats diabetes**	**27**	**100**	**63**	**100**
Yes	5	18.5	32	50.8
No	22	81.5	31	49.2
**TOTAL**	**27**	**100**	**63**	**100**
**Has blood hypertension**				
Yes	19	70.4	53	84.1
No	8	29.6	10	15.9
**TOTAL**	**27**	**100**	**63**	**100**
**Treats hypertension**				
Yes	17	63.0	36	57.1
No	10	37.0	27	42.9

Variables	Exposed		Not Exposed	
**TOTAL**	**27**	**100**	**63**	**100**
**Has hypertriglyceridemia**				
Yes	5	18.5	30	47.6
No	22	81.5	33	52.4
**TOTAL**	**27**	**100**	**63**	**100**
**Treats hypertriglyceridemia**				
Yes	2	7.4	21	33.3
No	25	92.6	42	66.7
**TOTAL**	**27**	**100**	**63**	**100**
**Has lithiasis**				
Yes	5	18.5	9	14.3
No	22	81.5	54	85.7
**TOTAL**	**27**	**100**	**63**	**100**
**Treats lithiasis**				
Yes	2	7.4	3	4.8
No	25	92.6	60	95.2
**TOTAL**	**27**	**100**	**63**	**100**
**Has UTI**				
Yes	12	44.4	22	34.9
No	15	55.6	41	65.1
**TOTAL**	**27**	**100**	**63**	**100**
**Treats UTI**				
Yes	9	33.3	15	23.8
No	18	66.7	48	76.2
**TOTAL**	**27**	**100**	**63**	**100**

UTI= Urinary Tract Infection; 1 x = one time; Source: the author.
